# RETRACTION: A Study of the Effect of Natural Brown Cotton Gauze on the Healing of Infected Wounds

**DOI:** 10.1111/srt.70220

**Published:** 2025-08-25

Jin S, Jin Z, Zhou W, Jin J, Sun Y, Jin Z. A study of the effect of natural brown cotton gauze on the healing of infected wounds. *Skin Res Technol*. 2024;30(6):e13778. doi:10.1111/srt.13778

The above article, published online on 4 June 2024 in Wiley Online Library (wileyonlinelibrary.com), has been retracted by agreement between the authors; *Skin Research and Technology*; and John Wiley & Sons Ltd. The article was accepted for publication following an insufficient peer review process that does not meet the standards of the journal's peer review policy. Further investigation by the publisher uncovered concerns regarding compliance with ethical standards for animal experimentation. Accordingly, the article has been retracted.

